# Novel β-Cyclodextrin and Catnip Essential Oil Inclusion Complex and Its Tick Repellent Properties

**DOI:** 10.3390/molecules26237391

**Published:** 2021-12-06

**Authors:** Jennifer Hogenbom, Mouaz Istanbouli, Nicoletta Faraone

**Affiliations:** Department of Chemistry, Acadia University, Wolfville, NS B4P 2R6, Canada; 131226h@acadiau.ca (J.H.); 140309i@acadiau.ca (M.I.)

**Keywords:** β-cyclodextrin, catnip, essential oils, nepetalactone, ticks, repellent bioassay, encapsulation, inclusion complex

## Abstract

Cyclodextrin inclusion complexes have been successfully used to encapsulate essential oils, improving their physicochemical properties and pharmacological effects. Besides being well-known for its effects on cats and other felines, catnip (*Nepeta cataria*) essential oil demonstrates repellency against blood-feeding pests such as mosquitoes. This study evaluates the tick repellency of catnip oil alone and encapsulated in β-cyclodextrin, prepared using the co-precipitation method at a 1:1 molar ratio. The physicochemical properties of this inclusion complex were characterized using GC-FID for encapsulation efficiency and yield and SPME/GC-MS for volatile emission. Qualitative assessment of complex formation was done by UV-Vis, FT-IR, ^1^H NMR, and SEM analyses. Catnip oil at 5% (*v*/*v*) demonstrated significant tick repellency over time, being comparable to DEET as used in commercial products. The prepared [catnip: β-CD] inclusion complex exerted significant tick repellency at lower concentration of the essential oil (equivalent of 1% *v*/*v*). The inclusion complex showed that the release of the active ingredient was consistent after 6 h, which could improve the effective repellent duration. These results demonstrated the effective tick repellent activity of catnip essential oil and the successful synthesis of the inclusion complex, suggesting that β-CDs are promising carriers to improve catnip oil properties and to expand its use in repellent formulations for tick management.

## 1. Introduction

Cyclodextrins (CDs) are important carrier molecules for the synthesis of different types of inclusion complexes. They are macrocyclic oligosaccharides composed of 6–8 glucose units to form a ring (named α-, β-, and γ-CD respectively). The unique structure based on a ring-shape gives cyclodextrins a hollow cone-like form with an open cavity. The dimension of the cavity ranges from 0.57 nm (α-CD) to 0.95 nm (γ-CD) resulting in a convenient size to encapsulate small organic molecules, including sesquiterpenes and diterpenes [1]. The arrangement of the OH and H groups is such that the central inner cavity is somewhat lipophilic, and the outer surface is hydrophilic. This makes cyclodextrins an ideal substrate to formulate inclusion complexes containing small hydrophobic active ingredients and improve their solubility in aqueous media. Examples of successful applications of the use of cyclodextrins include encapsulation substrates such as drugs, food products, and natural pesticides [2,3,4,5,6,7]. The synthesis of β-cyclodextrin inclusion complexes can be accomplished through methods such as co-precipitation [8,9], kneading [10,11], ultrasonication [12,13], freeze-drying [14], spray-drying [15], and the use of supercritical fluids [16]. Synthetic methods currently available are simple, require no (or limited amount of) organic solvents and no additional purification or cleanup steps, and use non-toxic materials, which makes the preparation of β-cyclodextrin inclusion complexes a desired and environmentally friendly process that falls into the green-synthesis approach [17,18].

Because of their chemical and physical properties, cyclodextrins have been used readily to encapsulate essential oils. Most of the components of essential oils are small organic molecules, usually less than 300 g/mol, such as monoterpenoids and sesquiterpenoids, which can be allocated inside the cyclodextrin cavity [19,20]. The encapsulation process is useful for counteracting some of the limiting properties of essential oils, expanding their application potential. On their own, these properties can make essential oils difficult to formulate consistently because of their volatility, low aqueous solubility, and sensitivity to degradation from UV exposure or oxidation [21,22]. Through cyclodextrin encapsulation, the volatile release of the active components is controlled and slowed down, improving product longevity. Moreover, when they are part of the inclusion complex, essential oil components are more stable and better protected from degradation by oxidation [23].

**Figure 1:**
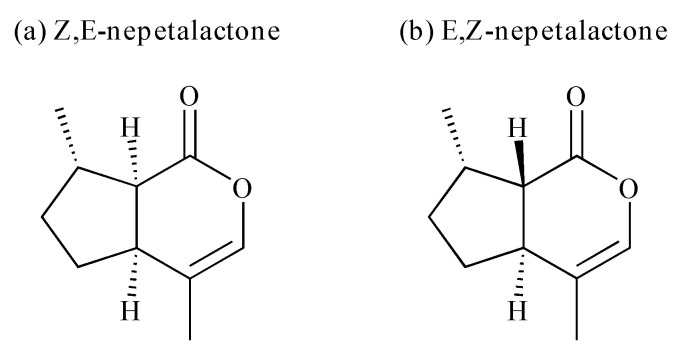
Structures of the two main nepetalactone isomers, *Z,E*-nepetalactone and *E,Z*-nepetalactone.

One of the highly interesting properties of essential oils is the ability to repel important pests such as those responsible for disease transmission. Many essential oils, including catnip (*Nepeta cataria* L.) essential oil, have demonstrated promising repellency against blood-feeding arthropods such as mosquitos and ticks. Catnip essential oil is generally dominated by nepetalactone isomers (Figure 1), but the compositions may vary depending on the phenological cycle and geographical location of the plant [24,25,26,27,28]. Catnip oil is one of the few essential oils that have been registered by the EPA as a skin-applied repellent ingredient [29]. Though there has been extensive research done on the repellency of catnip oil and its main component (e.g., nepetalactone) against mosquitos and other blood-feeding pests, very few studies have focused on the effects of this oil on ticks [30,31,32]. Synthetically modified (e.g., hydrogenated) catnip oil components were tested for repellency against mosquitos, stable flies, and ticks (*Ixodes scapularis* nymphs), with most of the results focusing on mosquitos [33]. In another study, the repellent activity of catnip oil and its components was tested against mosquitos, mites, and ticks (*Rhipicephalus appendiculatus*) [34]. Ticks are a major vector responsible for the transmission of pathogens that negatively impact humans. The northern spread of blacklegged ticks (*I. scapularis*), and the high incidence of tick-vectored Lyme disease cases reported in the past 5 years in North America has dramatically raised public awareness [35]. Lyme disease is a considerable public health concern, as it is the most common tick-transmitted disease in North America and Europe [36]. Additionally, rising average temperatures associated with climate change have been linked with the spread of *I. scapularis* ticks and increased rates of Lyme disease [37,38]. The key method for managing Lyme disease is through preventing tick bites, which emphasizes the importance of the development of effective repellent products.

In this study, inclusion complexes of catnip essential oil and β-cyclodextrin (β-CD) were prepared and characterized to determine inclusion complex formation and to assess the repellency against nymphal *I. scapularis* ticks. Non-encapsulated catnip essential oil was also tested for repellency against ticks to determine a range of effective concentrations. This study is the first, to our knowledge, to encapsulate catnip essential oil or its main component, nepetalactone, in a cyclodextrin complex. The formation of an inclusion complex can improve the feasibility of catnip essential oil as an effective tick repellent solution and minimize the negative qualities of the oil, such as its high volatility.

## 2. Results

### 2.1. GC-FID Quantification of Essential Oil in Inclusion Complexes

Catnip oil successfully encapsulated in the [catnip:β-CD] inclusion complex was determined by measuring through GC-FID the amount of oil extracted from the complex. Three different batches of inclusion complexes were prepared and analyzed. The mass concentration of surface oil and total oil, as well as the encapsulation efficiency (EE) and encapsulation yield (EY) were calculated (Table 1). The amount of surface oil concentration in the complexes was far less than the total oil concentration, which indicates that the encapsulation was highly efficient (>99%) and the overall oil measured in the complexes was contained within the inclusion complex. Therefore, negligible amounts of oil were adsorbed to the surfaces of the complexes. The average mass concentration of catnip oil in the inclusion complexes was 84 ± 2 μg/mg. The consistent results among the three batches indicates that the synthetic process is reproducible. The encapsulation yield was between 63% and 68% for the three prepared batches.

### 2.2. SPME/GC-MS Quantification of Volatile Release

**Figure 2:**
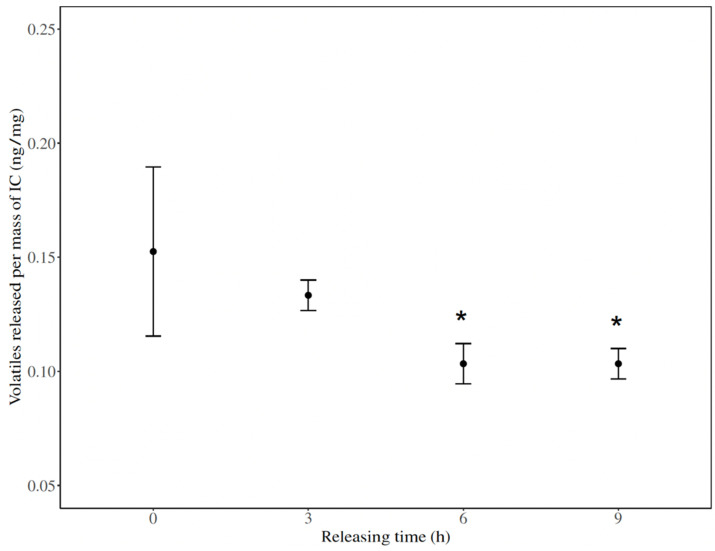
Release of catnip oil volatiles from [catnip: β-CD] inclusion complex (IC) over time. Volatiles measured by SPME/GC-MS (*n* = 3, * *p* < 0.05). Error bars represent the standard error for each time measured. Quantification of volatiles released was performed by using the standard curves of catnip oil in hexane. Asterisks indicate data points that are significantly different from time 0.

Catnip oil volatiles released from the inclusion complex over time were quantified by SPME/GC-MS (Table 2; Figure 2). The initial amount of released volatiles measured from the inclusion complex was 0.15 ± 0.06 ng/mg. The results showed that, after an initial decrease of the amount of catnip volatiles detected, it equalized at around 6 h and maintained a consistent volatile release of 0.10 ± 0.01 ng/mg.

### 2.3. FT-IR Analysis of Inclusion Complexes

**Figure 3:**
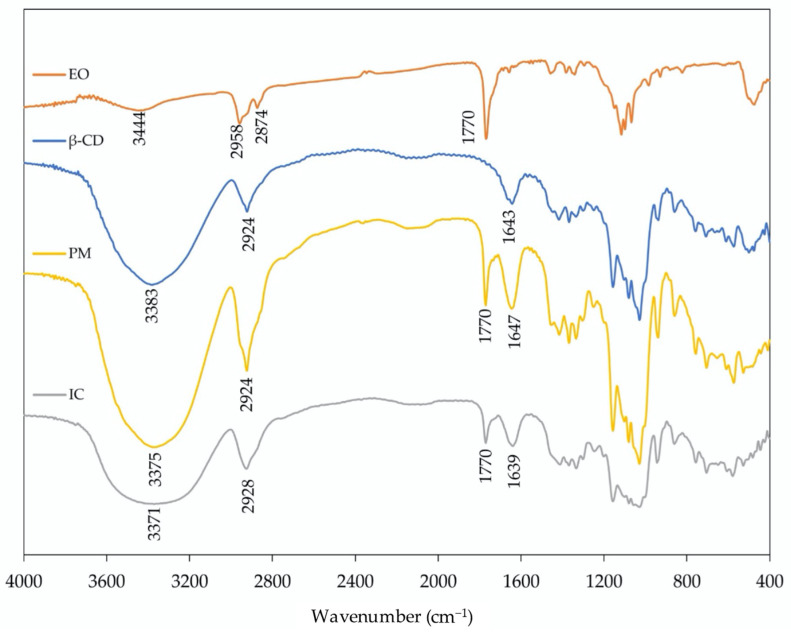
FT-IR spectra of catnip essential oil (EO), β-cyclodextrin (β-CD), their physical mixture (PM), and their inclusion complexes (IC).

FT-IR spectra were collected for catnip essential oil (EO), β-cyclodextrin, and their inclusion complexes and physical mixture (Figure 3). The spectra of the inclusion complex and physical mixture present common elements and demonstrate features from both the β-cyclodextrin and the catnip EO. Observed changes in shift, shape, and intensity of the FT-IR absorption peaks provided evidence for the formation of the inclusion complex. The catnip EO spectrum shows a band of C=O stretching (1770 cm^−1^) associated with the ketone group present in nepetalactone structure. This stretch is also present in both the inclusion complex and the physical mixture. The bands from C–H stretching in the catnip oil sample (2958–2874 cm^−1^) are not clearly visible in the inclusion complex and physical mixture due to the C-H stretching bands that are associated with the β-cyclodextrin (2924 cm^−1^). The β-cyclodextrin bands for O–H stretching (3600–3200 cm^−1^) and H–O–H bending (1642 cm^−1^) are also both visible in the inclusion complex and physical mixture spectra. The inclusion complex and the physical mixture present similar IR spectra. However, an overall broadening of the band shape (particularly at low frequencies, from 1200 to 400 cm^−1^) present in the spectra of the complex with respect to the corresponding physical mixture indicates a change in the hydrogen bonding as a consequence of the complexation [39].

### 2.4. NMR Spectroscopy

The results of the ^1^H NMR spectroscopy showed a change in the chemical shift for the protons on the inside of the β-cyclodextrin cavity (e.g., H-3 and H-5). This indicates that the chemical environment inside the cavity has changed as a result of the successful encapsulation of catnip oil inside the cyclodextrin cavity [9,40]. The chemical shifts of both β-cyclodextrin and the inclusion complex and their differences are shown in Table 3. Full spectra for both β-cyclodextrin and the [catnip: β-CD] IC are available in the Supplementary Information (Figure S1).

### 2.5. UV-Vis Spectroscopy

**Figure 4:**
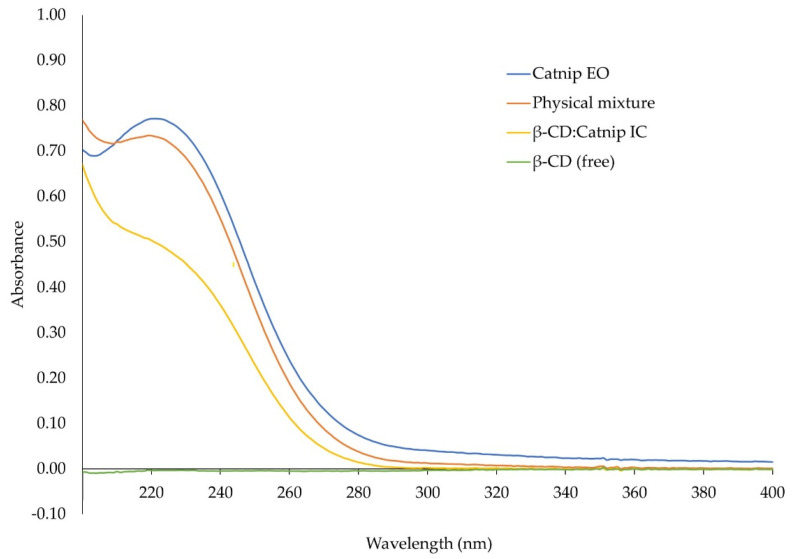
UV-Vis spectra of catnip essential oil (EO), β-cyclodextrin (β-CD), their inclusion complexes (IC), and their physical mixture.

The UV-Vis analysis of the catnip essential oil, [catnip:β-CD] inclusion complex, and the physical mixture of the essential oil and β-CD showed that catnip oil has a maximum absorbance at 220 nm; β-CD has no absorbance peaks in the measured range. The absorbance peak from the catnip oil decreased in the inclusion complex relative to both the physical mixture and the essential oil alone (Figure 4). This is the opposite of what was observed by Canbolat et al. [14] and may indicate a reduced solubility of the encapsulated oil as opposed to an increased solubility. It is also considered that this may be from the encapsulated oil being able to absorb less light while part of the inclusion complex.

### 2.6. Scanning Electron Microscopy (SEM)

**Figure 5:**
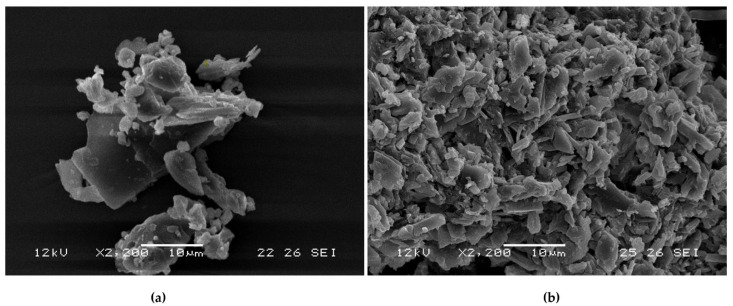
SEM images of (**a**) β-cyclodextrin and (**b**) [β-cyclodextrin:catnip oil] inclusion complex.

The morphology characteristics of the free β-CD starting material and the [catnip:β-CD] inclusion complex were examined through SEM (Figure 5). The structures of β-CD appeared as larger irregularly-shaped crystals with few smaller observable clusters. The inclusion complex were much smaller particles on average with some variation in size but more consistent than the β-CD. These observed structures are similar to previously reported results [9,41]. The changes in morphology reflect the processing of β-CD through the encapsulation method (i.e., co-precipitation) and drying.

### 2.7. Tick Repellency Bioassays

**Figure 6:**
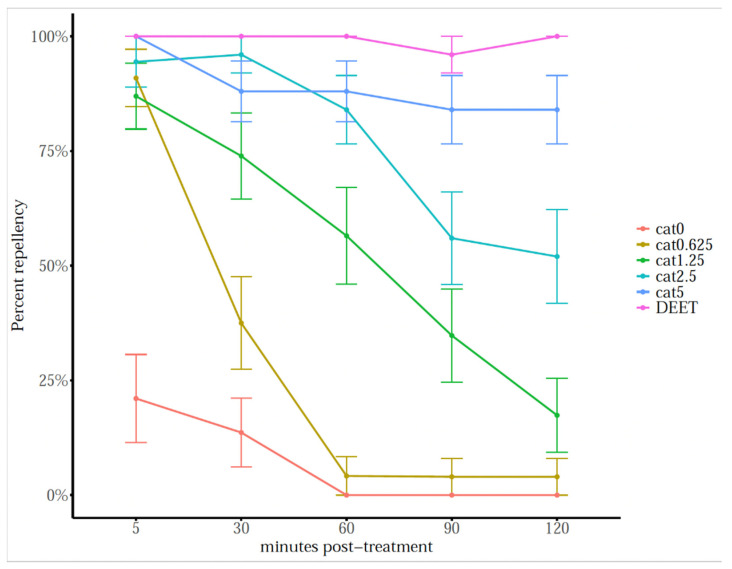
Mean percentage (±SEM) of ticks repelled by catnip essential oil at different concentrations and by DEET (25% *v*/*v*) in horizontal filter paper bioassays at different time points. *n* = 25 per concentration.

Catnip essential oil was effective at repelling ticks (F_5,78_ = 140.7, *p* < 0.001), and the repellent effect was significant over time (F_4,15_ = 33.0, *p* < 0.001), being effective up to 2 h (Figure 6; Appendix A Table A1). Catnip essential oil at 5% *v*/*v* was comparable to DEET (25% *v*/*v*) at repelling 84% (*z* = 1.872, *p* = 0.419) of ticks after 2 h post-application.

The complexation of the essential oil into the cyclodextrin cavity improved the solubility of the oil but reduced the hydrophilic nature of β-CD. In vertical bioassay, the [catnip:β-CD] inclusion complex was able to significantly repel ticks (Table 4) compared to the control (β-CD). Interestingly, 86 ± 6% of tested ticks were effectively repelled by the complex (*χ^2^* = 26.34, *p* < 0.001), indicating that the repellent properties of the catnip essential oil were retained when present in the inclusion complex.

## 3. Discussion

In this work, we have reported the use of catnip essential oil to synthesize an inclusion complex with cyclodextrins according to the co-precipitation method. To the best of our knowledge, this is the first example of catnip essential oil encapsulation using cyclodextrin as the wall material. The encapsulation of catnip essential oils has been previously attempted by preparing microcapsules using gelatin [42]. Previous work described the encapsulation of another iridoid compound (i.e., genipin) in β-cyclodextrin, reporting that the inclusion complex improved the stability and solubility in aqueous solution of the compound [43]. The type of cyclodextrin selected for this work was β-CD, based on the ease of availability and the ideal cavity size (i.e., inner diameter of 0.78 nm) being suitable for including the bicyclic structure of nepetalactone [1]. Modified cyclodextrins, such as hydroxypropyl-β-cyclodextrin, are also a valid alternative and may improve aqueous solubility compared to the natural cyclodextrins. However, the natural β-cyclodextrin represents a more appealing option as it has a lower cost and minimal toxicity and is more accessible, along with being suitable for the formation of inclusion complexes using the simple co-precipitation method [44].

The inclusion complexes in this study were prepared with a 1:1 molar ratio for β-cyclodextrin and catnip oil (or nepetalactone, as the main constituent and active ingredient). This molar ratio has been previously demonstrated to be effective for both other iridoid compounds (i.e., genipin [43]) and other monoterpenoids [6,40]. To determine the molar ratio, nepetalactone was used to approximate a molar amount of catnip oil. This can be done because the catnip essential oil used in this study contains 80% nepetalactone, with the other 20% being comprised of small (<1% each) amounts of other compounds, primarily other nepetalactonic compounds, monoterpenoids, and a few sesquiterpenoids (the chemical characterization of the oil provided by the supplier; see Materials and Methods).

The analyses performed on the inclusion complexes indicate that the encapsulation was successful. The extractions of surface and total oils from the inclusion complexes analyzed by GC-FID showed that there is no significant amount of oil adsorbed to the outer surface of the β-cyclodextrin, indicating that all the oil in the final product is included within the complex as expected. This is further supported by the ^1^H NMR results, which show that the protons on the inside of the cyclodextrin cavity have shifted up-field. The inclusion of the oil within the cyclodextrin cavity has a shielding effect on the H-3 and H-5 through intermolecular interactions [9,40]. The same change in chemical shift is not observed with other protons on the outside of the cyclodextrin as expected, since the surface oil extraction and analysis showed that there was not a significant amount on the outside of the cavity.

The prepared [catnip: β-CD] inclusion complexes seemed to exhibit an unexpected decrease in solubility compared to previously reported results [14]. This was seen both during sample preparation for UV-Vis analysis and the acquisition of UV-Vis results. Similarly, we observed a decrease in the solubility of β-CD when it was part of the [catnip: β-CD] inclusion complex during sample preparation for NMR analyses and bioassays. The inclusion complex did not readily dissolve in water or water/ethanol mixtures, even when gently heated, compared to the free β-CD. The fine powdery product was reported to form a suspension in water instead, which seemed relatively stable and took significant amounts of time to settle and separate completely. This phenomenon was already reported during the formation of inclusion complexes between β-CD and C10 aromatic molecules that caused a decrease of the solvent-accessible surface area, indicating that the inclusion complex has less solvent interaction with the surroundings [45]. It is important to note that the low aqueous solubility does not impact the product feasibility for use as a tick-repellent product; the inclusion complex performed well in the vertical bioassays and significantly repelled ticks. The change in solubility after inclusion complex formation may impact the potential application format. The [catnip: β-CD] complex may not be an ideal candidate for liquid formulations; however, it may be successfully incorporated in a cream-based lotion or for the synthesis of functionalized textiles [46].

The ability of catnip essential oil to repel ticks was assessed in horizontal bioassay. We have performed dose-response repellency trials with unencapsulated catnip oil to determine the effective concentration range. The results demonstrated that catnip oil is repellent against *I. scapularis* nymphs and that repellency follows a dose-response trend. To the best of our knowledge, this is the first reported example of catnip essential oil repellency studies on blacklegged ticks. The free catnip essential oils repelled 88% of tested ticks 1 h post application, and the repellent effect slightly decreased overtime, still repelling 84% of ticks after 2 h. Catnip oil and main components have been reported to repel blood-feeding pests such as stable flies [47], mosquitoes [31], and bed bugs [48], resulting in a valuable candidate for the development of effective pest-repellent products. This oil has been reported to repel *Aedes aegypti* L. (Culicidae) ten times more than DEET [28], and this ability is probably associated with the most effective constituent, nepetalactone.

In vertical bioassays, we tested the repellent action of the [catnip: β-CD] inclusion complexes against *I. scapularis* nymphs. Because the encapsulated product is in a solid form as opposed to the free catnip oil (liquid), we modified the bioassay method in order to uniformly apply the inclusion complex dissolved in ethanol/water. Horizontal bioassays were not suitable for the solid product due to the difficulty in distributing the product homogenously to a flat area (i.e., assay arena). Since the product was applied as a suspension in liquid, higher test concentrations were not homogeneously mixed due to the reduced solubility of the complex and the formation of a slurry that was difficult to apply. Based on the maximum amount of inclusion complex dissolved in the solution, we determined that the concentration tested was the equivalent of 1% (*v*/*v*) of catnip oil. Although the concentration of the active ingredient was low, the [catnip: β-CD] inclusion complex performed even better than the unencapsulated oil at similar concentrations, repelling 86% of tested ticks. The encapsulation of essential oils and other aromatic molecules has been reported to improve their physical properties, retaining or even enhancing their biological properties, such as exerting repellent action against arthropods [40,49]. Results from the SPME/GC-MS showed that the release of catnip volatiles from the [catnip: β-CD] inclusion complex reaches a plateau after 6 h, remaining consistent after an initial drop to about 60% of the initial amount. This demonstrates that encapsulation is a successful procedure in promoting a controlled release of volatile active ingredients over time. This desired outcome promotes the longevity of the product and will extend the effective time of tick-repellent action.

The use of cyclodextrins as successful carriers of essential oils and other botanical repellents offers novel avenues for the design of environmentally sustainable technologies to protect humans from disease vectors, such as ticks and mosquitoes. In addition, [cyclodextrin:essential oil] inclusion complexes offer the ideal substrate for the development of repellent textiles where active ingredients can be successfully immobilized and released under controlled conditions.

## 4. Materials and Methods

### 4.1. Chemicals

Catnip (*Nepeta cataria*) essential oil was purchased from New Direction Aromatics (Mississauga, ON, Canada). Ethyl alcohol anhydrous (EtOH) was purchased from Commercial Alcohols (Greenfield Global, Brampton, ON, Canada). β-cyclodextrin, hexanes, DEET (*N*,*N*-diethyl-meta-toluamide), and (−)-bornyl acetate were purchased from Sigma-Aldrich (Oakville (ON), Canada).

### 4.2. Preparation of Inclusion Complexes

A total of 0.0022 mol of β-cyclodextrin was dissolved in 100 mL of 1:2 ethanol:water while heated at 50–60 °C and under stirring conditions. Once fully dissolved, it was cooled to 30 °C. Approximately 0.0022 mol of catnip essential oil (determined from the main constituent, nepetalactone, MW: (166 g/mol) (0.0022 mol = 365 mg or 342 µL); density of catnip oil: 1.0663 g/mL) was dissolved in EtOH at 10% *v*/*v*. The catnip oil solution was added dropwise to the β-cyclodextrin solution and stirred for 30 min. The formed precipitate was collected by vacuum filtration and allowed to dry under vacuum for 1.5 h. The product was then dried further in an oven at 50 °C for 1.5 h before being transferred to a glass vial for storage and placed in a desiccator until further use [6]. Three batches of inclusion complexes were prepared separately and individually characterized to ensure product consistency.

### 4.3. GC-FID Quantification of EO in Inclusion Complexes

#### 4.3.1. Total Oil Extraction from Inclusion Complexes

Total oil contents were extracted from inclusion complexes in triplicate. For each extraction, 10 mg of powdered inclusion complex was measured into a 4 mL vial and dissolved in 1 mL of deionized H_2_O. To this, 1 mL of 1:1 hexane/ethanol was added, and the solution was shaken manually. Finally, 1 mL of hexanes was added, and the vials were sonicated for 30 min at room temperature and 40 kHz in an ultrasonic bath. The hexanes layer was collected, and the aqueous layer was extracted twice more with 1 mL of hexane for a total of 3 extractions. The organic layers were combined for each sample (final volume was 3.5 mL), dried with Na_2_SO_4_, and filtered using cotton wool [40].

#### 4.3.2. Surface Oil Extraction from Inclusion Complexes

Surface oils were extracted from each of the three prepared batches in triplicate. For each extraction, 90 mg of powdered inclusion complex was measured into a 4 mL vial with 2 mL of hexanes. The vial was vortexed for 1 min to extract surface oils and then the solution was filtered from the solid material. The extract was dried with Na_2_SO_4_ and filtered using cotton wool [50].

#### 4.3.3. GC-FID Analysis of EO Extracts

The assessment of encapsulation efficiency and encapsulation yield was performed as previously described [40]. Briefly, extracts were combined 1:1 with 80 ng/μL of (−)-bornyl acetate as an internal standard (final internal standard concentration 40 ng/μL) and analyzed by GC-FID. Each extract was analyzed on a gas chromatograph (Scion 450-GC; SCION Instruments UK Ltd., Livingston, UK) equipped with a flame ionization detector (FID). The GC-FID was equipped with a Rxi^®^-5silms capillary column (30 m × 0.25 mm, film thickness 0.25 mm; Restek Corporation, State College, PA, USA). The oven was programmed to start at 50 °C for 5 min, followed by a heating ramp of 7 °C/min to 220 °C, followed by 30 °C/min until 280 °C, where it was held for 5 min. The carrier gas was helium at a flow rate of 1.20 mL/min. The total analysis time was 36.29 min. The detector was set at 320 °C. The injector temperature was 250 °C; 1 μL of the sample was injected manually with a split ratio of 1:20. The quantification of EO content was done using a 10–400 ng/μL standard curve of catnip oil containing 40 ng/μL internal standard in hexanes. The main component in catnip oil, nepetalactone, has a total of 8 possible different isomers, with two that are usually considered the major components [32]. The catnip oil used in this study had 4 peaks that were identified as nepetalactone isomers, though they cannot be accurately assigned to specific isomers due to a lack of available standards. For the analysis performed in this study, the largest peak of the nepetalactone isomers was chosen and consistently used for the quantitation of catnip oil. The main nepetalactone peak from the catnip essential oil was normalized using the internal standard peak for quantification. The mass concentration of the essential oil in the inclusion complex was calculated along with the % EE and EY. The GC-FID data was used to determine the mass concentration of both the total (EO_t_) and surface (EO_s_) essential oils in the inclusion complexes. The encapsulation efficiency (EE) and encapsulation yield (EY) were calculated for the inclusion complexes using the following equations:

(1) $$\mathrm{EE}\left(\%\right)=\frac{{(\mathrm{EO}}_{t}-{\mathrm{EO}}_{s})}{{\mathrm{EO}}_{t}}\;\times \;100\%,\;$$

(2) $$\mathrm{EY}\left(\%\right)=\frac{{(\mathrm{EO}}_{t}-{\mathrm{EO}}_{s})}{{\mathrm{EO}}_{i}}\;\times \;100\%,$$

Using the measured EO_t_, EO_s_, and initial amount of essential oil used in preparing inclusion complexes (EO_i_) [51,52].

### 4.4. SPME/GC-MS Quantification of Volatile Release

The release of essential oil volatiles from the inclusion complexes was quantified using GC-MS by solid phase micro extraction (SPME) analysis as previously described [23,40,53]. The volatiles released were measured after 0, 3, 6, and 9 h of exposure at room temperature and 35–50% relative humidity. This was done by placing 20 mg of powdered inclusion complexes in open vials stored in a desiccator for the allotted time. After the time has passed, released volatiles were quantified by comparison to a liquid standard curve of catnip oil. SPME analysis was performed using a Scion SQ GC-MS equipped with a Gerstel multipurpose autosampler (MPS) (Gerstel, Mülheim an der Ruhr, Deutschland). The MPS allowed for automated SPME using a PDMS SPME fiber (100 μm). The SPME fiber was conditioned at 250 °C for 5 min before exposure to the sample. The samples in headspace vials were incubated for 10 min at 35 °C in the MPS agitator set at 250 rpm with an interval of 5 s on and 2 s off. The SPME fiber was exposed to the sample for 5 min with the same heating and stirring program. The SPME fiber was then desorbed in the GC inlet for 180 s before being reconditioned at 250 °C for 30 min. The same GC program described previously was used with a carrier gas flow rate of 1.00 mL/min, split ratio of 1:100, and split delay of 1 min. The liquid standard curve was quantified using the same GC-MS method with 1 μL liquid injections using Gerstel MPS.

### 4.5. FT-IR Analysis of Inclusion Complexes

Samples of the inclusion complexes, unencapsulated essential oil, free β-cyclodextrin, and a physical mixture were characterized with Fourier-transform infrared spectroscopy (FT-IR). The physical mixture was prepared by mixing catnip essential oil and β-cyclodextrin, in amounts proportional to those in the inclusion complexes, in a mortar and pestle briefly before analysis. Samples were prepared as KBr pressed pellets by mixing the samples with KBr powder in a ratio of ~1:10 and grinding in a mortar and pestle. The homogenized powder was then pressed into a disk with a hand press. The IR spectra were obtained in the range of 400–4000 cm^−1^ with a resolution of 3.857 cm^−1^ from an average of 32 scans on a FT-IR spectrometer (Nicolet Avatar 360, Nicolet Instrument Corporation, Danbury, CT, USA).

### 4.6. NMR Spectroscopy

The ^1^H NMR spectra for the inclusion complexes and free β-cyclodextrin samples in D_2_O were collected on a Bruker AVANCE 300 MHz spectrometer (Billerica, MA, USA) located at the Acadia Centre for Microstructural Analysis (ACMA) at Acadia University. NMR data acquisition and processing was done using TopSpin 2.1 (Bruker, Burlington, ON, Canada).

### 4.7. UV-Vis Spectroscopy

The inclusion complexes, catnip essential oil, free β-cyclodextrin, and a physical mixture (PM) were all measured by UV-Vis spectroscopy. Samples were prepared in DI H_2_O to have comparable concentrations of both the essential oil and β-cyclodextrin, using the mass concentration of essential oil in the inclusion complexes determined from GC-FID analysis. Samples were stirred for 24 h before absorbance was measured in the 190–400 nm range in quartz cuvettes with a path length of 1 cm on a Cary 100 Bio spectrophotometer (Varian, Palo Alto, CA, USA).

### 4.8. Scanning Electron Microscopy (SEM)

Samples were prepared for SEM analysis by mounting on SEM cylindrical specimen mounts using adhesive tabs. The mounted samples were coated with gold and palladium using a Polaron SC7640 Sputter Coater (Quorum Technologies, Lewes, UK) under automatic coating methods (Quorum Technologies, 2008). The SEM images were collected using a JEOL LV-5900 scanning electron microscope (JEOL USA, Peabody, MA, USA) located at the Acadia Centre for Microstructural Analysis (ACMA) at Acadia University.

### 4.9. Tick Repellency Bioassays

#### 4.9.1. Ticks

Naïve, unfed, mixed sex, nymphal *I. scapularis* ticks were used in horizontal and vertical repellency bioassays. Uninfected ticks were purchased from the Tick Rearing Facility Laboratory at Oklahoma State University (Stillwater, OK, USA). Ticks were stored on site in plastic containers lined with moistened Kimwipe^®^ in the fridge, at 4 °C in dark conditions. Nymphs were removed from the fridge and incubated at room temperature for at least 2 h prior to starting the bioassays. To confirm tick activity prior to performing vertical bioassays, ticks were placed on the center of a filter paper inside a Petri plate inside a marked “drop zone” (a 2.5 cm drawn circle); ticks that left the drop zone were determined as active.

#### 4.9.2. Treatments

In horizontal bioassays, treatments tested for tick repellency were catnip essential oils at 0.625, 1.25, 2.5, and 5% *v*/*v* in hexane. We used DEET at the concentration of 25% *v*/*v* as positive control. This concentration is commonly used in commercial pest repellent products. Hexane was the negative control. In vertical bioassays, [catnip: β-CD] inclusion complex was tested at 122 mg/mL in ethanol/water (1:1). Control was beta-CD only at 112 mg/mL (concentration adjusted to account for catnip oil contribution in inclusion complexes).

#### 4.9.3. Horizontal Bioassays

Catnip essential oil tick-repellent properties were assessed in horizontal bioassays. Behavioural experiments were performed as previously described in Faraone et al. (2019) [54]. Briefly, the behaviour of 5 nymphs per tested concentration (with 5 replication per treatment, *n* = 25) was monitored for 2 h. Ticks that did not cross the treated section were recorded as repelled at each different time point.

#### 4.9.4. Vertical Bioassays

To test the repellency of the inclusion complexes, we used a previously described vertical bioassay method [40] with some modifications. During initial control experiments without any repellents applied, it was observed that ticks did not show the expected natural behavioural tendency to climb up the cotton swab [55]; instead, they tended to walk downward most often. To follow this observed behaviour, the cotton swab was inverted so that tick would naturally move towards the tip of the cotton swab where the treatment was applied.

**Figure 7:**
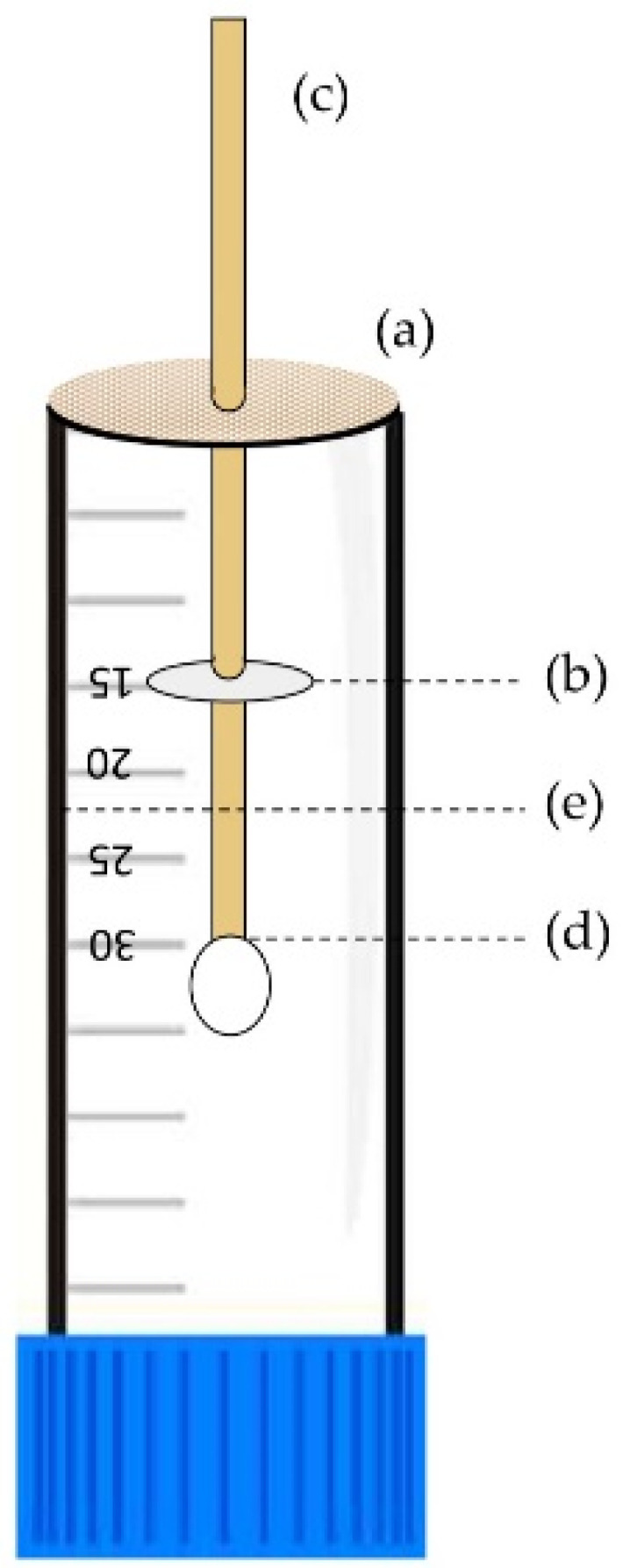
Vertical bioassay setup: the ‘v’ bottom of a Falcon tube was cut, and a mesh was hot glued over the opening (a). The filter paper with a thin layer of petroleum jelly was inserted 3 cm above the tip as a barrier and aligned with the 15 mL line of the falcon tube (b). A cotton swab was inserted through the tube and out the center of the mesh (c). The base of the cotton swab tip was aligned with the 30 mL marking on the tube and the treatment is applied evenly on the tip (d). The tick was released on the pencil marking 1.5 cm from the base of the cotton tip (e).

The bottom of 50 mL Falcon tubes (Fisher Scientific, Waltham, MA, USA) were cut and a mesh was glued over the opening. The center of the mesh was pierced to facilitate the insertion of the cotton swab (SolonCare^®^, Amd-Ritmed Inc., Montréal, QC, Canada). The wooden stems of the cotton swabs were marked 1.5 and 3 cm below the cotton swab tip using a pencil. Prior the experiment, prepared tubes were washed with soap and water followed by 70% *v*/*v* ethanol and dried fully. A diagram of the bioassay setup is shown in Figure 7.

The wooden stems of the swabs were submerged in water for one minute to absorb water and make them less electrostatic and improve tick mobility. Fifty (50) μL of the solution was pipetted uniformly on the cotton swab tip and left to dry for 3 min. A 1.5 cm diameter filter paper was cut, pierced through the center using pointed forceps, and a fine layer of petroleum jelly was applied on the surface of the filter paper. The cut filter paper was inserted through the wooden stick till the 3 cm marked line. Using forceps, the cotton swab was inserted through the tube and out the center of the mesh until the filter paper reached the 15 mL line of the Falcon tube.

Active ticks (i.e., questing) (*n* = 30) were individually positioned with a paintbrush to the 1.5 cm mark on the wooden stem of the cotton swab. Time was started when the Falcon tube was placed upright on its blue cap in a Plexiglas^®^ transparent box containing a humidifier to keep the humidity and temperature between 65–80% and 20–25 °C, respectively. Time was stopped when the tick reached the apex of the tip or dropped off the tip. Tick behaviour was monitored for up to 30 min. The observer was located one meter away to minimize the introduction of uncontrolled variables from the observer such as scent, breath, and shadows.

#### 4.9.5. Statistical Analysis

Statistical analysis was performed by using RStudio Version 1.1.453 (RStudio Team 2018, Vienna, Austria). The mass of EO extracted from inclusion complex and essential oil volatile emission were analyzed using a linear mixed-effect model (lmer). After assessing the significance of the model using the ANOVA function, we performed a post-hoc pairwise comparison of interaction using the emmeans function (emmeans package) on the model to determine the differences between means produced by different treatments. Results from different time points were independently analyzed. In horizontal bioassays, repellency was analyzed with generalized linear mixed effect regression (glmer) with a binomial link to model the logit of tick repelled, followed by simultaneous tests for general linear hypotheses and multiple comparisons of means (Tukey’s test). Vertical bioassay data with not normal distribution were subjected to non-parametric tests (i.e., Kruskal-Wallis), followed by a post-hoc test (i.e., Dunn test) to compare the different treatments. Differences were considered significant at *p* < 0.05.

## Figures and Tables

**Table 1:** Determination of total catnip essential oil (EO) concentration extracted from the inclusion complex and concentration of catnip essential oil recovered from inclusion complex surface, with associated standard error (SE). Report of encapsulation efficiency (EE) and encapsulation yield (EY). Analyses were performed by GC-FID. *n* = 3.

Batch	Surface Mass Concentration EO ± SE (μg/mg)	Total Mass Concentration EO ± SE (μg/mg)	EE (%)	EY (%)
1	0.170 ± 0.008	87 ± 3	99.8	68
2	0.280 ± 0.031	83 ± 4	99.7	64
3	0.265 ± 0.004	81 ± 1	99.7	63

**Table 2:** Release of catnip oil volatiles from [catnip: β-CD] inclusion complex (IC) over time. Volatiles measured by SPME/GC-MS (*n* = 3). Quantification of volatiles released was performed by using the standard curves of catnip oil in hexane.

Time (h)	Volatiles Released per Mass of IC			Percentage of Initial Volatiles
	ng/mg (±SE)	t	*p*	
0	0.15 (±0.04)	-	-	-
3	0.13 (±0.01)	−1.746	0.1	88%
6	0.10 (±0.01)	−3.073	0.02	68%
9	0.10 (±0.01)	−3.073	0.02	68%

**Table 3:** Chemical shift (δ) and corresponding changes (Δδ) between the ^1^H-NMR spectra of β-CD and the [catnip: β-CD] inclusion complex (IC). Protons are labelled according to the β-CD structure.

	δ β-CD (ppm)	δ [catnip: β-CD] IC (ppm)	Δδ (ppm)	β-CD Structure
H-1	5.063	5.061	−0.002	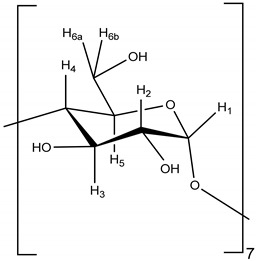
H-2	3.642	3.640	−0.002	
H-3	3.960	3.949	−0.011	
H-4	3.576	3.575	−0.001	
H-5	3.845	3.830	−0.015	
H-6	3.873	3.867	−0.006	

**Table 4:** Mean percentage (±SEM) of ticks repelled by β-cyclodextrin-catnip complex in vertical bioassays (*n* = 30).

Treat	Repellency (±SEM)		
	%	*χ^2^*	*p*
β-CD-catnip	86 (±6)	26.34	<0.001
β-CD	20 (±4)	-	-

## Data Availability

Raw data will be available upon request.

## Supplementary Materials

The following are available online, Figure S1: ^1^H NMR of B-CD: CATNIP and ^1^H NMR of FREE B-CD.

## Sample Availability

Samples of [catnip: β-CD] inclusion complex are available from the authors.

## Appendix A

Table A1 provides the results from the horizontal repellency bioassay.

**Table A1:** Mean percentage (±SEM) of ticks repelled by catnip essential oil at different concentrations and by DEET (25% *v*/*v*) in horizontal filter paper bioassays at different time points. *n* = 25 per concentration.

Treat		Repellency (±SEM)%						
	% *v*/*v*	5 min	30 min	60 min	90 min	120 min	*z*	*p*
catnip	0.0	21 (±1)	14 (±7)	0 (±0)	0 (±0)	0 (±0)	-	-
	0.625	90 (±6)	38 (±10)	42 (±4)	4 (±4)	0 (±0)	8.417	<0.001
	1.25	87 (±7)	74 (±9)	57 (±11)	35 (±10)	17 (±8)	7.971	<0.001
	2.5	94 (±5)	96 (±4)	84 (±7)	56 (±10)	52 (±10)	8.394	<0.001
	5	100 (±0)	88 (±7)	88 (±7)	84 (±7)	84 (±7)	9.452	<0.001
DEET	25	100 (±0)	100 (±0)	100 (±0)	96 (±4)	100 (±0)	9.266	<0.001
