# Supplementary material for: Novel β-Cyclodextrin and Catnip Essential Oil Inclusion Complex and Its Tick Repellent Properties
Source: Molecules. 2021 Dec 6;26(23):7391. doi: 10.3390/molecules26237391 (PMC8659168; doi:10.3390/molecules26237391)
Supplement: Supplementary file 1 [file molecules-26-07391-s001.zip › molecules-1450324-supplementary.pdf]

1H NMR of B-CD:CATNIP

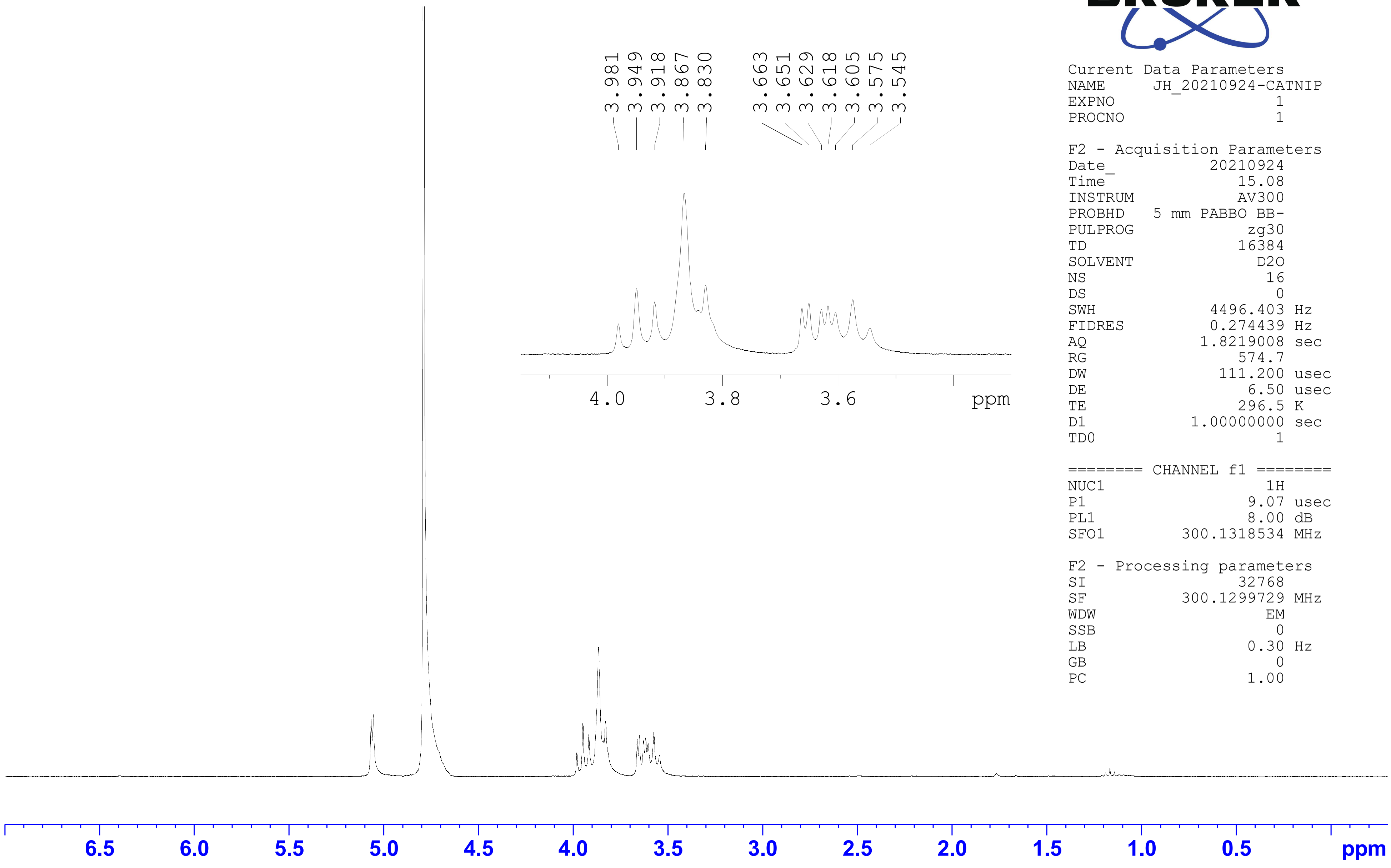


```
Current Data Parameters
NAME       JH_20210924-CATNIP
EXPNO                       1
PROCNO                      1

F2 - Acquisition Parameters
Date_                20210924
Time                    15.08
INSTRUM                 AV300
PROBHD    5 mm PABBO BB-
PULPROG                  zg30
TD                      16384
SOLVENT                   D2O
NS                         16
DS                          0
SWH                  4496.403 Hz
FIDRES               0.274439 Hz
AQ                  1.8219008 sec
RG                      574.7
DW                    111.200 usec
DE                       6.50 usec
TE                      296.5 K
D1                 1.00000000 sec
TD0                         1

======== CHANNEL f1 ========
NUC1                       1H
P1                       9.07 usec
PL1                      8.00 dB
SFO1              300.1318534 MHz

F2 - Processing parameters
SI                      32768
SF                300.1299729 MHz
WDW                        EM
SSB                         0
LB                       0.30 Hz
GB                          0
PC                       1.00
```

1H NMR of FREE B-CD

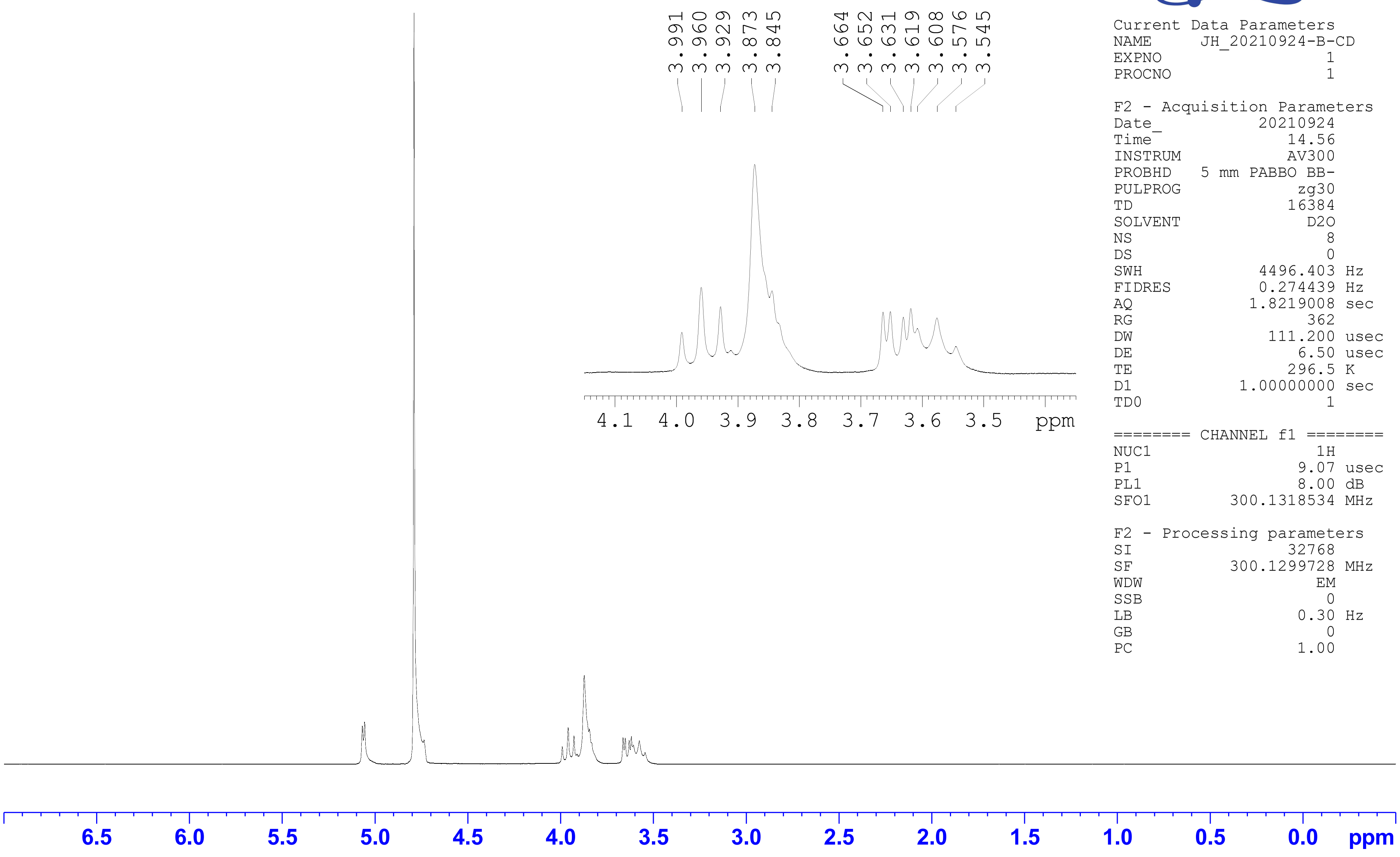

Current Data Parameters
NAME JH_20210924-B-CD
EXPNO 1
PROCNO 1

F2 - Acquisition Parameters
Date_ 20210924
Time_ 14.56
INSTRUM AV300
PROBHD 5 mm PABBO BB-
PULPROG zg30
TD 16384
SOLVENT D2O
NS 8
DS 0
SWH 4496.403 Hz
FIDRES 0.274439 Hz
AQ 1.8219008 sec
RG 362
DW 111.200 usec
DE 6.50 usec
TE 296.5 K
D1 1.00000000 sec
TD0 1

======== CHANNEL f1 ========
NUC1 1H
P1 9.07 usec
PL1 8.00 dB
SFO1 300.1318534 MHz

F2 - Processing parameters
SI 32768
SF 300.1299728 MHz
WDW EM
SSB 0
LB 0.30 Hz
GB 0
PC 1.00
